# Retraction Note: Paediatric Ewing-like sarcoma arising from the cranium – a unique diagnostic challenge

**DOI:** 10.1186/s13000-016-0549-4

**Published:** 2016-10-11

**Authors:** Ian J. Robertson, Fadel Bennani, Ronan S. Ryan, Waqar Khan, M. Kevin Barry

**Affiliations:** 1 Department of Surgery, Mayo General Hospital, Castlebar, Co Mayo Ireland; 2 Department of Histopathology, Mayo General Hospital, Castlebar, Co Mayo Ireland; 3 Department of Diagnostic Radiology, Mayo General Hospital, Castlebar, Co Mayo Ireland; 4 Professor of Surgery, National University of Ireland, Galway (NUIG), Galway, Ireland

## Retraction Note

This article [1] has been retracted by the authors because, contrary to the statement in the article, they did not obtain the necessary written informed consent from the patient’s parents to publish this case. The article is no longer available online in order to protect the patient’s privacy. The authors have agreed to the retraction.
